# Behaviour change communication for control of tuberculosis by healthcare workers in DOTS facilities in Nigeria

**DOI:** 10.11604/pamj.2020.36.306.21640

**Published:** 2020-08-19

**Authors:** Evelyn Nwanebe Nwagu, Lawreta Ijeoma Abugu, Wamanyi Yohanna, Dorothy Nwakaego Eze, Amaka Harry Ononuju, Agatha Nneka Obayi

**Affiliations:** 1 University of Nigeria Nsukka, Department of Human Kinetics and Health Education, Nsukka, Nigeria,; 2 Adamawa State College of Health Technology, Department of Community Health, Michika, Nigeria,; 3 Nsukka Health Centre, Nsukka, Enugu state, Nigeria

**Keywords:** Behaviour change communication, control of tuberculosis, healthcare workers, knowledge, beliefs, practice

## Abstract

**Introduction:**

prevention and control of tuberculosis (TB) should be behaviour-centred to facilitate change of unhealthy behaviours that encourage the spread of the infective agent. This study aimed to ascertain the knowledge, beliefs and practices of Behavior Change Communication (BCC) in the control of TB by healthcare workers in DOTS in Nigeria.

**Methods:**

using the qualitative research approach, we interviewed 38 healthcare workers from two states in Nigeria. The interview questions consisted of 13 open-ended questions framed to elicit information about the health workers' knowledge, beliefs and practices of BCC in the control of TB. Analysis was done using the conventional content analysis.

**Results:**

the following themes emerged: lack of knowledge and understanding about BCC; BCC believed to be important in the control of TB; lack of adequate skills for BCC; some aspects of BCC practiced; BCC was mainly done in health facilities; and lack of adequate system for maintaining long term change.

**Conclusion:**

the BCC training needs of healthcare workers in the area of study have been revealed. These should form the basis for effective BCC capacity building programme for healthcare workers in the prevention and control of TB. We recommended that BCC should go beyond interpersonal communication to community-wide campaign through mass media to produce a massive change in behaviour that will enable the elimination of TB.

## Introduction

Many disease conditions among people nationwide occur as a result of unhealthy lifestyles arising from ignorance and faulty traditional beliefs. Through health communication, healthcare workers can inform the public about important health issues, provide correct health information and correct misconceptions about health matters. Health communication has been recognized as a key strategy to inform the public about health concerns and to maintain important health issues on the public agenda [1]. Health communication however should go beyond providing information on health issues to bringing about positive change in behaviour. Well designed health communications are known to change people's knowledge, attitudes, and behaviours by increasing risk perception, reinforcing positive behaviors, influencing social norms, pointing to available social support and health services and empowering individuals to change or improve their health conditions [2]. Prevention and control of tuberculosis (TB) should be behaviour-centred to facilitate change of unhealthy behaviours that encourage the spread of the infective agent. By using appropriate Behavior Change Communication (BCC), healthcare workers can bring about the needed behaviour change for elimination of TB. The goal of BCC is to promote healthy behaviours and reduce risk taking. Behavior change health communications are very important in disease prevention, health promotion and the overall quality of life of individuals. BCC encompasses health communication, social and community mobilization, and it evolved from information, education and communication (IEC) strategies [3]. Strategic use of BCC should apply targeted messages and tailored approaches to promote healthy behaviours and reduced risk taking [3].

While appropriate communication improves clients´ satisfaction, compliance to health instructions and positive health outcomes; a poorly done communication can lead to misunderstanding and non compliance to health instructions. Health Educators therefore need to be knowledgeable and skillful in identifying appropriate communication strategies that bring about behavior change with subsequent health promotion. The components of BCC range from interpersonal communication between a community health worker and her client to multi-level mass media campaigns. BCC interventions are an integral part of all types of health promotion and disease prevention, and have been shown to significantly improve behaviours, particularly in health promoting practices [3,4] and disease prevention [5]. In Nigeria like in many other African countries, health promotion programmes are often compromised by the rise in the health sector of donor-funded programmes focused on therapeutic interventions for specific diseases such as HIV/AIDS, TB and malaria [6] to the detriment of preventive and health promoting interventions. Suffice it to say that many logistical and medical components of the global response to TB are relatively robust but the communication part is not [7]. Although a cure for TB has been available for more than 50 years, it has been observed that the DOTS (Directly Observed Treatment, Short-course) - the strategy recommended by the World Health Organization (WHO) for fighting the disease, and which is used internationally - did for a long time not carry a communication component [7]. It was only in 2005 that a communication component was incorporated into DOTS, and many countries are yet to effectively incorporate the communication component [8]. The need to incorporate the BCC into the DOTS strategy in Nigeria is quite urgent to address the high burden of TB in the country. In 2017, it was reported that 87% of new TB cases occurred in the 30 high TB burden countries and eight countries including Nigeria accounted for two thirds of the new TB cases [9]. This study aimed to ascertain the knowledge, beliefs and practices of BCC in the control of TB by healthcare workers in DOTS facilities before planning a context specific and effective BCC capacity building programme for the health workers.

## Methods

We used the qualitative research approach to ascertain the BCC knowledge, beliefs and practices among healthcare workers in all DOTS facilities in Nigeria. Two states (Enugu state and Kaduna state) were randomly sampled from all the states in Nigeria for this study. Purposive sampling of 38 participants from the health facilities in selected states (20 participants from Kaduna and 18 participants from Enugu) was done. Data for the study were collected from the selected 38 healthcare workers in the DOTS facilities in two states of Nigeria using individual, semi-structured interviews. At the point at which these 38 individuals had completed the interviews, the research team conferred to determine if additional recruitment was needed; however, the interviewer indicated that saturation had been reached. Thus, a total of 38 healthcare providers served as the sample for this study. The demographic characteristics of participants are shown in Table 1. Informed written consents of the participants were obtained prior to the interview. Participation in the study was completely voluntary. All interviews were anonymous. The interviews were conducted face-to-face in private and quiet locations. The interview questions consisted of 13 open-ended questions framed to elicit information about the health workers' knowledge, beliefs and practices of BCC in the control of TB. The semi-structured interview guide was developed by the study team and pilot tested through three mock interviews. The guide was revised accordingly after the mock interviews. The interview began with structured questions regarding healthcare workers demographic information - gender, age, profession, qualification, location and length of service. This was followed by questions on: what participants know about BCC; the BCC skills they have; their beliefs about the importance of BCC in the control of Tuberculosis; participants' application of BCC skills in the control of tuberculosis; their ease or difficulty in using BCC in the control of tuberculosis; their beliefs about the outcome of using BCC in their control of TB infection; who they get involved in BCC in their control of TB; what each person involved in BCC for control of TB do; when, where, how often and with who they engage in such activities; how long changes take to occur; how they know when a change in behaviour has occurred; the systems they use for maintaining long term change; and what they will suggest to facilitate BCC in TB control.

**Table 1:** demographic characteristics of participants

Variable	Frequency		Total	Percent
	**Enugu State**	**Kaduna State**		
**Gender**				
Male	12	4	16	42.1
Female	6	16	22	57.9
Total	18	20	38	100.0
**Age**				
25 - 35	2	5	7	18.4
36 - 45	4	7	11	28.9
45 and above	12	8	20	52.6
Total	18	20	38	100.0
**Profession**				
Nursing	10	5	15	39.5
CHEW	2	6	8	21.0
JCHEW	2	3	5	13.2
Lab technician	0	2	2	5.3
CHO	3	4	7	18.4
Medical doctor	1	0	1	2.6
Total	18	20	38	100
**Qualification**				
Diploma	5	19	24	63.2
B.Sc	8	1	9	23.7
M.Sc	5	0	5	13.2
Total	18	20	38	100.0
**Location**				
Private	1	0	1	2.6
General Hospital	8	2	10	26.3
Health Center	9	18	27	71.1
Total	18	20	38	100.0
**Length of service**				
less than 5 years	1	0	1	2.6
5 -10 years	4	6	10	26.3
11 - 20 years	4	8	12	31.6
21 years and above	9	6	15	39.5
Total	18	20	38	100.0

The interview responses were digitally recorded and transcribed verbatim. Quantitative data were entered into SPSS. Descriptive analyses were conducted. Qualitative analysis was done using the framework method - an approach within a broad family of analysis methods often termed thematic analysis or qualitative content analysis [10]. All personal identifiers were removed and the transcripts were imported into the open code software version 4.03 [11] a software program for analysing qualitative data. The study was conducted with 38 healthcare workers in the study area. As shown in Table 1, more than half of the study participants were females (57.9%) and aged 45 years and above (52.6%). Greater proportion of the participants were nurses (39.5%), had diploma (63.2%), work in health centres (71.1%) and have been in DOTS for 21 years and above (39.5%). The responses were reviewed and coded to identify the initial categories. The initial set of codes were expanded and refined to better reflect the common elements that existed across the full data set. Patton [12] asserted that this iterative style of analysis is a recognized characteristic of the qualitative research method. Patterns were identified inductively by analyzing codes associated with specific purposes of the study. Further coding refinement occurred when all the codes associated with a particular interview question were examined for convergence. The qualitative content analysis was then used to identify themes across the codes. Examination of the response patterns across the data sets ultimately led to the emergence and identification of themes reflective of healthcare workers' knowledge, beliefs and practices of BCC in the control of TB.

**Ethical clearance:** ethical approvals for the study was granted by the Research Ethics Committee Faculty of Education University of Nigeria (REC/FE/2018/000002) and by the state ministry of health (MOH/ADM/744/VOL.1/541).

## Results

Six main themes emerged from the study, namely: lack of knowledge and understanding about BCC; BCC believed to be important in the control of TB; lack of adequate skills for BCC; some aspects of BCC practiced; BCC was mainly done in health facilities; and lack of adequate system for maintaining long term change.

**Knowledge of BCC by healthcare workers**

**What healthcare workers know about BCC:** participants were asked what they know about BCC. Almost every participant mentioned that BCC has to do with communicating with patient and relatives about TB. A couple of participants went further to add that BCC should be done through health education, counseling, home visits, mass media, and outreach programme. Few of the healthcare workers also mentioned that such skills as; patient rapport and acceptance are needed for effective BCC. Some participants merely equate BCC to health instruction and health talk while some simply take it for counseling. Example a female CHO described BCC as “...Just to tell them to be taking their drugs daily...” PK10.

**Lack of knowledge and understanding about BCC:** responses of some participants show total lack of knowledge and understanding of BCC. For instance; when asked “what do you know about BCC” PE23 a female CHEW said “behavior change communication is a system by which in the community, like if you see a patient with some of the diseases that can be infected, then you start to behave somehow like stigma, like avoiding the person...” Although many of the participants said that they know what BCC is, the response of some of them to further questions show that they are not conversant with BCC. For example when asked “how do you know when a change in behaviour has occurred?”, a female JCHEW responded “merely seeing the person I know that a change has occurred” PK12.

**Beliefs about BCC**

**BCC believed to be important in the control of TB:** all the participants believed that BCC is important in the control of tuberculosis.

**Healthcare workers´ perceptions about the ease or difficulty of applying BCC:** most participants believed that BCC is easy. PK3 said “...yes it is quite easy. I explain to the patient how important it is to take their drug”. Few participants believe BCC is difficult. A male participant (PE33) in responding to the question “How easy or difficult do you find using BCC in the control of tuberculosis?” said “... in short, it ee well it is difficult in a way because... they are predominantly farmers and illiterates (referring to his clients). Most of them don´t adhere to what you teach ... actually it is difficult. But with those who are literate, it is less difficult” PE28 noted that “ sometimes it is very easy with some people, for some patients it may be very difficult for you to convince them to accept the sickness and then may be adhere to the...”

**Perceived outcomes of BCC:** when asked “What do you think would happen if you engage in BCC in your control of TB infection?” Many of the participants believe TB infection will be reduced if BCC is practiced.

**Variations in when and how of BCC:** participants had varied views about when to conduct BCC. Many said any time, some said as need arise, yet some others believed BCC should be started when a patient test positive to TB. When asked how long changes take to occur, many participants believed that it depends on the patient. While it will take some time in some patients, it takes only a little time in some other patients. Factors which they attributed to the length of time it take before change include literacy, adherence to treatment and the type of change.

**Practices of BCC**

**Some aspects of BCC practiced:** behaviour change communications which many of the participants engage in include: Health education/health instructions. Much of such education was centred on encouraging the patients to take their drugs appropriately. Other things they educate patients and care givers on were; proper feeding, avoidance of stigmatization and proper disposal of sputum. PE24 stated “... apart from the drugs they take, we teach them about their behaviour as individuals, like you don´t... if you have tuberculosis you don´t cough and throw away the sputum indiscriminately, you dispose it very well because it is from the sputum that others can get the infection they are told ee how to dispose the sputum, how to care for themselves and also other people around.” PE28 equally stated “...I try to let the person know that TB can be cured, letting the person know how to take his drugs, adhere to his drug regimen, educate the care givers to accept the person and render necessary care to him ensuring that he is taking his drug and that he is being well fed”.

**Health facility and community based practices:** when participants were asked when, where, how often and with whom they engage in BCC activities, many participants indicated that they carry out the BCC in the health facilities during follow up visits. Some also stated that they engage in home visits. Many participants indicated that they engage family and community members on volunteer basis. All the healthcare workers in the DOT units and other healthcare workers were also involved. For instance PE25 stated “... we get involved the DOTs focal persons, the laboratory, all the people that work in the TB unit.”

**Practices for sustaining change:** when asked the systems they have for maintaining long term change, many participants indicated that they do not have any system for maintaining long term change. Very few participants however stated that they use such practices as; continuous counseling, follow up, education and home visiting to maintain long term change.

**Perceived action needed to facilitate BCC:** when asked to suggest what can be done to facilitate BCC in TB control, up to half of the participants suggested workshops and seminars. A few others suggested free provision of facility for good practice and increase in campaign.

## Discussion

In this research study, we qualitatively examined healthcare workers´ knowledge, beliefs and practices of BCC in the control of TB in DOTS facilities in Nigeria. Findings showed that although many participants possessed basic knowledge of BCC, they lacked indebt understanding of what constitutes BCC. Some of the participants merely equated BCC to health instruction and health talk. Similarly, the participants in a study conducted to appraise midwives observance of Behaviour Change Communication Programme for Maternal and Child Health in Kaduna state equates BCC to health talks [13]. It is important to note that BCC is not same as routine communication as BCC target specific health condition and it is tailor-made for variable groups thus it assists in enabling a supportive environment which indirectly assists members of society not only to initiate but even sustain positive and desirable behaviour outcomes [14,15]. Behaviour Change Communication interventions have been identified as a crucial aspect of any health-promotion and disease prevention programme, which have been shown to significantly improve behaviours, particularly in health promoting practices [3,4]. Healthcare workers, therefore, need to be quite knowledgeable about the techniques and strategies of BCC to be able to use it in the control of TB. A lot of the eventual outcome of the BCC strategy depends upon the knowledge level of the healthcare worker and their efficacy to share knowledge with the target population [16]. Health communications through health instruction and talks alone are not adequate to bring about positive change in behavior of individuals and communities. With respect to control of TB, advocacy, communication and social mobilization are three distinct sets of activities, all of which have the shared goal of bringing about behavioural change [17]. Any BCC programme should, therefore, encompass health communication, social and community mobilization. Community participation has been recognized as a basic principle of BCC programs [18]. Healthcare workers need to understand the crucial role of community members in BCC particularly in the control of TB. Such an understanding will enable them to engage community members and caregivers in their BCC efforts.

The healthcare workers attitude to BCC did not march their practice of BCC. While all the participants believed that BCC is important in the control of tuberculosis, not all of them practiced BCC. There are several possible reasons for this; some healthcare workers perceived BCC to be a difficult task, many lacked adequate knowledge and skill in BCC. The attitudes and beliefs of healthcare workers play important roles in the patients´ healthcare experiences. In addition to having an impact on their interaction with their patient, HCWs´ attitudes and beliefs can also influence their motivation to make changes to their own practices and behaviors, as well as how they perform their jobs and their desire to continue as a member of the healthcare workforce altogether. Thus, targeting HCW attitudes through BCC programmes can both improve quality of care and have a long-term impact on the strength of the healthcare workforce as a whole [19]. Participants rightly perceived that TB infection will be reduced if BCC is practiced. BCC interventions have been shown to significantly improve behaviours, particularly in health-promoting practices [3,4] and disease prevention [5]. The process of BCC varied among participants. Many said any time, some said as need arise, yet some others believed BCC should be started when a patient test positive to TB. When asked how long changes take to occur, many participants believed that it depends on the patient. While it will take some time in some patients, it takes only a little time in some other patients. Factors which they attributed to the length of time it takes before change include literacy, adherence to treatment and the type of change.

Only a few aspects of BCC was carried out by healthcare workers, mainly; Health education/health instructions. Much of such education was centred on encouraging the patient to take their drugs appropriately. In a similar study, Akin-Otiko and Bhengu observed that BCC for Maternal and Child Health were poorly implemented in all the facilities in Kaduna state as the midwives lacked the capability for BCC [13]. Healthcare workers are often introduced into the workforce lacking the knowledge and skills necessary to perform their assigned responsibilities. Adequate pre-service training should create the essential foundation for providing quality health services, while training insufficiencies can have a widespread impact on a worker´s capacity to address the needs of the community [19]. Apart from proper pre-service training, regular refresher and in-service education opportunities are necessary to ensure that HCWs have retained and are adhering to earlier training, and are updated on healthcare advancements [20]. Most BCC efforts were carried out in the Health facility and only a few community-based practices were done. Effective BCC programme is not limited to interpersonal communication but should also include the use of mass media and community mobilization [3]. Traditional communication tools, as well as the modern communication technology, have been shown to be effective tools for communication. Many participants indicated that they do not have any system for maintaining long term change. Very few participants however stated that they use such practices as; continuous counseling, follow up, education and home visiting to maintain long term change. Continued implementation of effective interventions and sustained enabling social environments is crucial to a sustained positive impact of BCC. Behavior change is a dynamic process and as such it requires time from contemplating the behavior to achieving maintenance and needs to be reinforced over longer periods of time [21].

**Limitations of the study:** like in every qualitative study, this study has limitation. The findings cannot be generalized to the entire healthcare workers in the country. However, the study provided an in-depth knowledge of healthcare workers´ knowledge, beliefs and practices of BCC for TB control which can be effectively used to plan a training programme on BCC for healthcare workers in the study area.

## Conclusion

Healthcare workers in DOTS facilities in the study area do not have adequate knowledge of BCC. They believe BCC to be important in the control of TB but they lack the skill to implement BCC adequately. The findings have revealed the BCC training needs of healthcare workers in the area of study. These should form the basis for effective BCC capacity building programme for healthcare workers in the prevention and control of TB. We recommend that BCC should go beyond interpersonal communication to community wide campaign through mass media to produce a massive change in behavour that will enable the elimination of TB.

### What is known about this topic

- Health communication has long been recognized and documented by the World Health Organization (WHO) as a key strategy to inform the public about health concerns and to maintain important health issues on the public agenda;
- The Directly Observed Treatment, Short-course (DOTS) - the strategy recommended by the World Health Organization for fighting the Tuberculosis, and which is used internationally-did for a long time not carry a communication component;
- It was only in 2005 that a communication component was incorporated into DOTS, and many countries are yet to effectively incorporate the communication component.

### What this study adds

- Healthcare workers in DOTS facilities in Nigeria do not have adequate knowledge of Behaviour Change Communication (BCC);
- Healthcare workers in DOTS facilities in Nigeria believe that BCC is important in the control of TB but they lack the skill to implement BCC adequately.
